# Supplementary material for: LTA4H extensively associates with mRNAs and lncRNAs indicative of its novel regulatory targets
Source: PeerJ. 2023 Mar 10;11:e14875. doi: 10.7717/peerj.14875 (PMC10010175; doi:10.7717/peerj.14875)
Supplement: File S2 [file peerj-11-14875-s005.zip › Raw data files/QRT-PCR validation experiment/Primer information.docx]

| NEAT1-ip-F | CTGTGGACTGTTGAGTTACT |
| --- | --- |
| NEAT1-ip-R | ATGCGGTATAGCCTTATCTAC |
| COL7A1-ip-F | GGAGCCATACCTTCACTC |
| COL7A1-ip-R | CCTTACCTTGCCTCTGTC |
| ROR2-ip-F | GTGTCTCTGCGTGTCTTG |
| ROR2-ip-R | ACACAGAAACACACCAACA |
| LTBP3-ip-F | CCTCCTCAGCCTATACCAA |
| LTBP3-ip-R | TCCTAGTGATGCCTTCCTT |
| HSP90B1-ip-F | TTGGATCTTGCTGTGGTT |
| HSP90B1-ip-R | CTGAGGCGAAGCATTCTT |
| LINC00657-ip-F | GCATACATCAATGGCTATCA |
| LINC00657-ip-R | TCAGGACTTGTTCATTAGGT |
| EGFR-ip -F | GATACTCATAACCTGCTAACAC |
| EGFR -ip-R | GTATGTGTCTGTGTATGGTATG |

primer binding site F primer： AATTAACCCTCACTAAAGGG
primer binding site R primer： GTCCTTATCATCGTCGTCTT
